# Girls’ explanations for being unvaccinated or under vaccinated against human papillomavirus: a content analysis of survey responses

**DOI:** 10.1186/s12889-015-2657-6

**Published:** 2015-12-22

**Authors:** Alice S. Forster, Jo Waller, Harriet L. Bowyer, Laura A. V. Marlow

**Affiliations:** Health Behaviour Research Centre, Department of Epidemiology & Public Health, UCL, Gower Street, WC1E 6BT London, UK

**Keywords:** Vaccination, Papillomavirus Vaccines, Ethnicity, race, uptake, Adolescent

## Abstract

**Background:**

In England HPV vaccination is offered to all girls age 12–13 years, free-at-the-point-of-receipt, mostly in schools. Coverage is good, but around 20 % of girls remain unvaccinated. This research sought to explore reasons for being un-/under vaccinated.

**Methods:**

An ethnically diverse sample of girls aged 15–16 years attending one of twelve London schools completed a survey three years after being offered HPV vaccination. Girls reported their HPV vaccine status and those who were unvaccinated (had not received any doses of the vaccine) or under vaccinated (had not completed the recommended 3-dose course) recorded reasons for their un-/under vaccinated status. Reasons were reported using free-text and content analysis was used to analyse responses.

**Results:**

Around 74 % of un-/under vaccinated girls provided a reason for their vaccination status (n = 259). Among unvaccinated girls, the most common reasons related to lack of perceived need for vaccination, concerns about safety and lack of parental consent. Girls who were under vaccinated gave practical reasons, including the need for more information (e.g. not knowing that multiple doses were needed), administrative issues (e.g. school absence), health and procedural concerns (e.g. fear of needles). Descriptively, there were few differences in the reasons given between girls from different ethnic backgrounds. Girls from Black and Asian backgrounds more commonly thought that the vaccine was not needed. Lack of parental consent without providing further explanation was most often cited by girls from Black backgrounds.

**Conclusions:**

Safety concerns and lack of perceived need should be addressed to encourage informed uptake of HPV vaccination. Immunisation programme coordinators may be able to increase series completion by tackling practical problems facing under vaccinated girls.

## Background

In 2008, vaccination against human papillomavirus (HPV) was recommended in the United Kingdom (UK) for 12 and 13 year old girls. The vaccine protects against the two oncogenic HPV types most strongly associated with cervical cancer (HPV 16 and 18) and is over 99 % effective at preventing precancerous lesions associated with these virus types [1, 2]. In the UK, quadrivalent HPV vaccine is currently delivered almost exclusively through schools. A three-dose schedule over a six month period was used from 2008 until September 2014, when a two-dose schedule was adopted. Uptake of HPV vaccination in England is high overall (90 % for the first dose and 80 % for all three doses in 2013/2014), but uptake varies widely across England [3].

Understanding the reasons why some girls remain un-/under vaccinated will help to identify targets for information campaigns or wider policy changes that can help establish and maintain high coverage across all areas. To date, only a few, mainly qualitative studies have explored the reasons for being un-/under vaccinated among girls in England [4–8]. Girls in these studies generally lacked knowledge about the vaccine and were unclear about why it was needed, largely because of the novelty of the vaccine and because they did not feel at risk of HPV infection. Some studies have also highlighted that physical discomfort may be a barrier to girls completing the vaccination series [6, 7]. In other countries with school-based vaccination programmes, similar themes have been identified [9].

Research exploring the association between socio-demographic characteristics and uptake of HPV vaccination at a population-level has suggested that girls from some ethnic minority backgrounds are less likely to receive HPV vaccination than girls from White backgrounds [10, 11]. This remains the case when controlling for deprivation [12]. In particular, certain areas of the UK with large ethnic minority populations appear to have the poorest uptake. In London, where the population is highly ethnically diverse [3], mean uptake is 76 % and is as low as 67 % in some areas. No studies have explored why this might be. A review of studies conducted before HPV vaccination was introduced suggested that mothers from ethnic minority backgrounds were unsure of the need for HPV vaccination, particularly for 12–13 year olds [13].

Often research exploring attitudes towards HPV vaccination has been conducted with parents, for example [14–16]. However, because uptake of HPV vaccination in the UK is high [17], studies of British parents are often comprise samples of parents who intend to or have accepted HPV vaccination for their child [18–20]. Parents who actively decline the vaccine are likely to be over-represented in the ‘unvaccinated’ group. Identifying non-accepting parents, particularly those from ethnic minority backgrounds, and those who may not have actively engaged with the vaccine offer, is therefore a research challenge. However, evidence suggests that girls are also involved in the decision-making process [4, 21], and in the class-room context recruitment is likely to reflect a more representative sample of those who are un-/under vaccinated. In the present study we therefore qualitatively assessed reasons for being un-/under vaccinated among a sample of girls (aged 15 to 16 years) from a diverse range of ethnic backgrounds.

## Methods

### Participants

Participants were girls aged 15 to 16 years old who completed a questionnaire three years after their year group were offered HPV vaccination in school (when they were 12 or 13 years). Participants were recruited through schools. To recruit the schools we created a sampling frame using Free School Meal Eligibility (FSME) [22] and General Certificate in Secondary Education (GCSE) attainment as indicators of socio-economic position. We categorised schools as being above or below the national average for FSME (23 %, [23]) and GCSE (53 % of pupils attaining 5 grades A*-C; [24]). All London government-funded schools with female pupils were entered into the sampling frame if they reported HPV uptake within 10 % of the national average [25] (89 schools). We randomly contacted schools in each cell of the sampling frame until the target sample was recruited (n = 13 schools). Data were collected over two waves in 2011/12 and 2012/13. One week before the research took place, parents received an information sheet about the study and an opt-out form. Consent was implied if the opt-out form was not returned. All girls in attendance when the research was conducted were given information about the study. Consent was presumed if the girls completed the questionnaire. We chose an opt-out consent approach to maximise the response rate, while still giving parents and girls the option of opting out (to enhance the validity of the findings). The study was assessed to be low risk (as it was a questionnaire study and was not asking questions that might invoke distress), asking 15 and 16 year old students about their attitudes to vaccination and vaccination behaviour. The schools had viewed the questionnaires and study protocol and assessed this approach to be appropriate. Researchers gave an introductory talk to the students emphasising that participation was voluntary and anonymous and that they did not need to complete any questions they felt uncomfortable answering. The approach was approved by the ethics committee. All girls received a debrief sheet after completing the questionnaire. More information about the sampling method is provided elsewhere [11, 26].

### Measures

During a school lesson, respondents completed the questionnaire which asked them to report their HPV vaccine status. Vaccination status was assessed by asking girls which best applied to them (‘I have had all three doses of the HPV vaccine’; ‘I have had 1 or 2 doses of the HPV vaccine’; ‘I have been offered the HPV vaccine, but I haven’t had it’; ‘I have not been offered the HPV vaccine’ or ‘I don’t know’). Those who had not accepted the vaccine or who had started, but not completed the course were asked to report their reasons using free-text (‘If you have not had the HPV vaccine, or you didn't have all 3 doses, why was this?’). Girls were 12 or 13 when they were offered the HPV vaccine and parental consent would have been requested for vaccination (although not mandatory), so girls’ responses are likely to be a combination of their own beliefs about the vaccine and their parents’ reasons for not consenting. Participants reported their ethnicity, religion, and whether they were practising that religion and completed the Family Affluence Scale [27], a four item, objective measure of family affluence. The present study focuses on the reasons girls gave for being un-/under vaccinated, but the questionnaire also assessed HPV knowledge, attitudes to vaccination and sexual behaviour, the results of which are published elsewhere [11, 26]. The study was approved by the University College London research ethics committee (0630/002).

### Analysis

Participants who had received one or two doses of the HPV vaccine (as opposed to the recommended 3 doses) were classified as under vaccinated and those who had been offered the vaccine, but who had not received any doses were classified as unvaccinated.

Chi-square (significance p < 0.05) was used to test for demographic differences in whether girls provided a reason to explain their vaccination status and to test for demographic differences between unvaccinated and under vaccinated girls who had provided a reason for their vaccination status. We used content analysis to analyse the girls’ free-text responses. After reading through the responses LM developed a coding frame. Responses were then coded by LM and AF with an inter-rater reliability of 0.86 (kappa). Any discrepancies were discussed and resolved. Where multiple reasons were given multiple codes were allocated.

Girls’ reasons are reported and discussed by vaccination status. We explored girls’ reasons by ethnic group, but numbers were too small for statistical comparison.

## Results

### Sample characteristics

Over two waves of data collection 2,163 girls completed the questionnaire (78 % of those registered at the schools). After excluding those who did not know or did not report their vaccine status or who reported that they had not been offered the vaccine (309 girls, 14.3 %), 19.2 % (355/1,854) had not been fully vaccinated. These 355 girls had either received one or two doses of the HPV vaccine (under vaccinated) or had been offered the vaccine but had not had it (unvaccinated). The present analyses include data from 259 of these girls (74 %) who provided a reason for their un-/under vaccinated status. Of these, 202 (78 %) were unvaccinated and 57 (22 %) were under vaccinated (comprising girls from all 12 of the participating schools). The majority of included participants provided one sentence answers to the free-text question.

Girls were mostly from White (31 %), Black (29 %) or Asian backgrounds (20 %). Around 2 % did not provide their ethnicity and just under a fifth of girls were from an ethnic background other than White, Black or Asian; mostly mixed backgrounds. In terms of religion, 42 % were Christian, 24 % were Muslim, 24 % had no religion, 9 % had another religion and less than 1 % did not provide their religion. Of girls who reported having a religion, 66 % said they were practising. Mean affluence score was 5.49 (SD = 1.84, range 1–10). Providing a reason was not significantly associated with ethnicity, practising a religion or family affluence, but was associated with religion (Table 1). Girls who were Christian or Muslim were less likely to provide a reason than girls from other religions or those reporting no religion. Among individuals who provided a reason, there were no demographic differences between those who were under vaccinated and those who were unvaccinated.

**Table 1:** Sample characteristics of un-/under vaccinated girls (n = 355)

	Reason not given^a^	Reason given^a^ and unvaccinated	Reason given^a^ and under vaccinated
	(n = 96)	(n = 202)	(n = 57)
Ethnicity			
White	23 (24.0)	61 (30.2)	18 (31.6)
Black	37 (38.5)	64 (31.7)	12 (21.1)
Asian	21 (21.9)	34 (16.8)	17 (29.8)
Other	14 (14.6)	39 (19.3)	10 (17.5)
Missing	1 (1.0)	4 (2.0)	0 (0)
Religion			
None	11 (11.5)	48 (23.8)	14 (24.6)
Christian	54 (56.3)	89 (44.1)	20 (35.1)
Muslim	27 (28.1)	45 (22.3)	17 (29.8)
Other	4 (4.2)	19 (9.4)	5 (8.8)
Missing	0 (0)	1 (0.5)	1 (1.8)
Practising religion ^b^			
Yes	48 (56.5)	100 (65.8)	28 (65.1)
No	36 (42.4)	49 (32.2)	14 (32.6)
Missing	1 (1.2)	3 (2.0)	1 (2.3)
FAS ^c^			
Low affluence	68 (70.8)	132 (65.4)	40 (70.2)
High affluence	23 (24.0)	67 (33.2)	15 (26.3)
Missing	5 (5.2)	3 (1.5)	2 (3.5)

^a^ Reason to explain girls’ vaccination status

^b.^ Among those who reported having a religion

^c.^ FAS = Family Affluence Scale

### Reasons for being un-/under vaccinated

For unvaccinated girls the most common reasons given to explain their vaccination status were lack of parental consent (41 %, 82/202), safety concerns (25 %, 51/202) and believing that they did not need the vaccination (19 %, 38/202). For under vaccinated girls, the main reasons for their vaccination status were practical problems, including administrative problems (51 %, 29/57, for example, being absent from school on the day of vaccination) and needing more information (11 %, 6/57). Health reasons (9 %, 5/57, for example, existing health conditions perceived to be contraindications for vaccination) and procedural reasons (5 %, 3/57, for example, fear of needles) were also given by a few of these girls. Reasons offered by girls themselves and their beliefs about their parents’ decisions are provided in Table 2 and by ethnic group in Table 3. Quotes provided below are accompanied by the participants’ self-reported ethnicity and participant number in parentheses.

**Table 2:** Reasons provided by unvaccinated and under vaccinated participants to explain their vaccination status

Major theme (subthemes)	Examples	n (%) Unvaccinated	n (%) Under vaccinated
		n = 202	n = 57
Lack of parental consent (without explanation)	*My parents don’t want me to get it*	30 (14.9)	1 (1.8)
Lack of parental consent (with explanation)	Provided below under major themes	52 (25.7)	3 (5.3)
Safety concerns (concern about side effects or long term effects, the novelty of the vaccine, wanting more research, seeing press reports about the death of a girl from HPV vaccine, prefer to delay vaccination)	*I was scared about the long-term effects as the vaccine hasn't been around for long* *My Mum felt it was as if we were being tested on*	51 (25.3)	2 (3.5)
The vaccine isn’t needed (not sexually active, not planning on being sexually active, no history of cervical cancer in the family, don’t need it)	*Because I am not sexually active and will not be until I get married* *My Mum didn’t think it was necessary for me to have the vaccine since I won’t be sleeping around*	38 (18.8)	1 (1.8)
Administrative reasons (being absent from school, moving schools, being out of the country, recent migration, not having a consent form, didn’t want the vaccine in school, not offered vaccination doses).	*I wasn’t in school the day the 3rd injections happened * *My parents …preferred me to have it at the doctors, not school.*	26 (12.8)	29 (50.9)
Need for more information (was not aware of the vaccine, didn’t understand it, not enough information)	*I didn’t know about it* *My Mum wasn’t sure what it was*	8 (4.0)	6 (10.5)
Procedural issues (afraid of or dislike needles, pain)	*I’m scared of needles*	22 (10.9)	3 (5.3)
General vaccination beliefs (don’t believe in vaccinations, don’t’ believe in manmade treatments)	*…I wouldn’t want a man-made treatment* *Mum didn’t think it was natural to have it*	5 (2.5)	0 (0)
Health reasons (existing health condition, got ill after previous dose, allergic to ingredients, unwell when vaccine offered).	*I have not had it because I suffer from other conditions and therefore was more likely for me to have a negative reaction.* *My Mum… thinks I might be allergic to HPV vaccine*	9 (4.5)	5 (8.8)
Other reasons (don’t know/can’t remember, didn’t want it with no explanation, other)	*Because I didn’t want to*	22 (10.9)	11 (19.3)

Note: Column percent may not be equal 100 % as multiple reasons were given by participants.

**Table 3:** Most common reasons for being un-/under vaccinated against HPV within each ethnic group (n = 255)

	Ethnicity n (column %)			
	White (n = 79)	Black (n = 76)	Asian (n = 51)	Other (n = 49)
Lack of parental consent (without explanation)	5 (6.3)	15 (19.7)	7 (13.7)	4 (8.2)
Lack of parental consent (with explanation)	22 (27.9)	17 (22.4)	6 (11.8)	9 (18.4)
Safety concerns	21 (26.6)	18 (23.7)	6 (11.8)	8 (16.3)
The vaccine isn’t needed	11 (13.3)	13 (17.1)	11 (21.6)	4 (8.2)
Administrative reasons	18 (22.8)	13 (17.1)	13 (25.5)	11 (22.5)
Need for more information	4 (5.1)	5 (6.6)	1 (2.0)	4 (8.2)
Procedural issues	12 (15.2)	5 (6.6)	1 (2.0)	6 (12.2)
General vaccination beliefs	1 (1.3)	0 (0)	0 (0)	3 (6.1)
Health reasons	5 (6.3)	1 (1.3)	3 (5.9)	5 (10.2)
Other reasons	6 (7.6)	10 (13.2)	10 (19.6)	5 (10.2)

#### Lack of parental consent

Among unvaccinated girls, the most commonly cited reason for their vaccination status was parental refusal, cited by 41 % (82/202) of girls. Around two thirds of these girls gave a reason for their parents’ refusal (63 %, 52/82), while a third offered no explanation (37 %, 30/82). Parental concerns were rarely mentioned by the girls who were under vaccinated.

Concordance between girls’ and parents’ decision making was evident for some cases.

*“My mother and I decided I should have the vaccine when I was older”* (Mixed White/ Asian, TS099).

In other cases there were conflicts between parents and daughters, and even between parents.

*“My mother didn't want me to have it, even though I did”* (Black Caribbean, BC351)

*“I didn't have it because my mum didn't agree with having lots of vaccines but my dad was fine with it. But I didn't have it in the end”* (Mixed White/Black Caribbean, HPS314).

There were also examples of girls who disagreed with their parents’ reasoning, but still remained unvaccinated.

*“My parents objected because of the death of one girl who was given the vaccine, even though it was a different batch and she had underlying medical conditions”* (Black Caribbean, WHS277).

#### Safety concerns

Unvaccinated girls reported having concerns about the safety of the vaccine (25 % 51/202), but this was only expressed by two under vaccinated girls (4 %, 2/57), who presumably felt the vaccine was safe enough for them to have at least one dose. Many of the unvaccinated girls’ concerns related to the novelty of the vaccine, and fear that vaccination could cause unforeseen side-effects.

*“The repercussions of the vaccination are not known. Long term side effects may accumulate over the year. We are the 'guinea pigs' this is being tested on”* (Mixed White/Asian, BC109)*.*

Some of these girls said that their parents did not trust that sufficient research had been done to guarantee the safety of the vaccine. Others said that they would be allowed to have it in the future.

*“My mum didn't trust the vaccine because it was new”* (Turkish, HF085).

*“My family wanted to wait for further research”* (Mixed White/Black Caribbean, ES293).

#### The vaccine isn’t needed

Feeling that the HPV vaccine was unnecessary was another frequently cited reason for being unvaccinated (19 %, 38/202). Some felt this was because of the sexually transmitted nature of HPV.

*“Because I am not sexually active so I wouldn’t need it”* (Mixed Black Jamaican/Asian, WHS217)

*“Because I am not going to have sex before marriage”* (Pakistani, TS137)

Others felt that being the first generation to have HPV vaccination, a perception of low prevalence of HPV or a lack of family history meant it was unnecessary.

*“My mother never had it, so I didn't need it”* (Black Caribbean, RF208)

*“The cancer looked very rare, cancerous diseases don't run in my family”* (White British, MA048).

Unvaccinated girls also reported that their parents thought the vaccine was not necessary because their daughters would not have multiple sexual partners or because their mothers did not have the vaccine themselves and had not developed cervical cancer. Many girls simply said that their parents did not think the vaccine was necessary.

*“Mother didn’t believe it was necessary…, recently migrated here, HPV is not common in Africa”* (Black African, BTG055)

*“My mum did not think it was necessary for me to have the vaccine since I won’t be sleeping around” (*Filipino*,* BC116*).*

#### Administrative reasons

Administrative issues were the reasons most frequently reported by under vaccinated girls (51 %, 29/57), but were also reported by a small proportion of unvaccinated girls (13 %, 26/202). These issues were not related to a decision not to vaccinate, but were explanations for missing doses for practical reasons. School absence was cited by 19 girls, 13 reported that they did not want to have the vaccine in school (some preferred to have it with their family doctor), 13 said that they had not returned the vaccination consent form, seven girls reported that they had recently moved schools and missed the vaccination being offered and three reported that they were not in the country.

*“I never got round to having the 3rd one [dose] because I switched schools”* (Indian, TS091)*.*

*“I never brought back the letter because I had lost it and when I had it signed it was too late”* (Black Caribbean, RF211).

### Need for more information

Around 11 % of girls who were under vaccinated reported that their vaccination status was a result of needing more information (6/57). In particular, these girls did not understand that multiple doses of the vaccine were needed. This was not cited by as many unvaccinated girls (only 4 % reported this, 8/202), suggesting that they or their parents felt they had sufficient information to decide not to receive the vaccine. Although under vaccinated girls and their parents may have received enough information to obtain the first or second vaccination dose, girls were not aware that they needed to obtain further doses.

*“Not sure. I don’t understand it well enough”* (Mixed White/Black Caribbean, HPS231)

*“Don’t know, the school only offered 2 [doses]”* (Polish, BTG040)

#### Procedural issues

Fear of needles and dislike of injections were reported by around 11 % of unvaccinated girls (22/202), and a smaller proportion of under vaccinated girls (5 %, 3/57).

*“I hate needles”* (Mixed White/Black Caribbean, CEB025)

#### General vaccination beliefs

Having generally negative beliefs about vaccinations was reported by a few unvaccinated girls (3 %, 5/202), with all of these girls believing that their parents’ beliefs about vaccination in general influenced their decision to withhold their consent for the HPV vaccine.

*“Mum didn’t think it was natural to have it”* (White British, TS198)*.*

#### Health reasons

Existing health conditions, including epilepsy and organ transplantation, were cited by a few unvaccinated girls, as well as some girls believing they were allergic to ingredients in the vaccine (5 % of unvaccinated girls, 9/202, and 9 % of under vaccinated girls, 5/57). Some under vaccinated girls had reactions following early doses of the HPV vaccine, which they attributed to the vaccine and decided not to finish the course.

*“After the first vaccine, I started to feel lighted headed, also fainted a few times, doctor told me not to complete it”* (Mixed White/Black Caribbean, HF045)*.*

#### Other reasons

Around 11 % of unvaccinated girls (22/202) and 19 % of under vaccinated girls (11/57) did not provide a clear explanation for their vaccination status other than that they did not want the vaccine or that they could not remember their own or their parents’ decision making process.

### Reasons for being un-/under vaccinated by ethnic group

Among girls from White ethnic backgrounds, lack of parental consent (with explanation) was the most frequently cited reason for being un-/under vaccinated (28 %), but it was also often cited by girls from Black (22 %) and Other ethnic backgrounds (18 %; and less so among girls from Asian backgrounds, 12 %). Safety concerns were frequently cited by girls from White (26 %), Black (24 %) and Other ethnic backgrounds (16 %), but less commonly among girls from Asian backgrounds (12 %). Administrative reasons were the most commonly cited reason among girls from Asian (26 %) and Other ethnic backgrounds (23 %), but also often mentioned by girls from White backgrounds (23 %). Girls from Black backgrounds were likely to cite lack of parental consent without providing further explanation (20 %) and girls from Asian backgrounds commonly stated that they (or their parents) did not think the vaccine was needed (22 %), although this was also mentioned by a notable proportion of girls from Black backgrounds (17 %).

## Discussion

This study explored girls’ reasons for being un-/under vaccinated against HPV in a free-at-the-point-of-receipt school-based HPV vaccination programme. Based in London, the sample included girls from a diverse range of ethnic backgrounds. The reasons for being unvaccinated and being under vaccinated appeared to differ. Unvaccinated girls cited lack of parental consent, their own and their parents’ safety concerns about a new vaccine and the perception that they did not need the vaccine. Conversely, under vaccinated girls were more likely to cite practical problems to explain their vaccination status, including administrative issues (e.g. school absence), needing more information (e.g. that multiple doses were required), health and procedural concerns (e.g. fear of needles). In general, girls from different ethnic backgrounds cited similar reasons to explain their un-/under vaccinated status. However there was some suggestion that girls from Black and Asian backgrounds (or their parents) more commonly thought that the vaccine was not needed. Girls from Black backgrounds were most likely to report lack of parental consent without providing further explanation.

Girls in this study were 12 and 13 when they were offered HPV vaccination and among unvaccinated girls, parents played an important role in the decision to be vaccinated. In particular, these girls perceived that their parents were concerned about the novelty of the vaccine and potential unknown long-term side effects, something that has been expressed in previous research [15, 28, 29]. Some unvaccinated girls reported that they or their parents believed that the vaccine was not needed and this was commonly cited by girls from Black and Asian backgrounds. This echoes parents’ previously reported concerns about vaccinating their daughters against a sexually transmitted infection when they are not yet sexually active [30] and has previously been expressed by mothers from ethnic and religious minority groups [15, 28].

Parental concerns about vaccination were rarely reported by girls who had initiated but not finished the vaccination course, but were reported by girls who had not started the series, whereas under vaccinated girls reported reasons that related to the girls’ own behaviour (e.g. being absent from school). This suggests that parents may influence vaccination receipt and girls may influence series completion. Interventions to address HPV vaccination uptake may benefit from targeting parents for the initial dose of the vaccination series and adolescents for future doses. There is evidence that immunisation nurses are already chasing-up under vaccinated girls [31], however future efforts to facilitate girls completing the vaccination course is likely to increase the workload of immunisation nurses further [32]. Now that the vaccination course has been changed to two doses only, practical problems may become less of an issue. Needing more information was only important to under vaccinated girls, suggesting that current vaccination information materials are considered sufficient by many girls and their parents. However, for girls who were under vaccinated, it appears that ensuring awareness of the number of doses required would be beneficial. Presumably this is only an issue for girls who were absent from school on vaccination days, as those with consent to receive the vaccination and who were present in school, would have been offered it during the school day. Immunisation programme coordinators may wish to address this in information resources targeted at girls who miss vaccination doses.

There are a number of limitations to this study. Girls were asked to respond about the HPV vaccine three years after they had been offered it, so recall bias may have occurred in girls’ recollection of their HPV vaccine status or the reason for their vaccination status. Review of medical records would have been the gold standard for assessing vaccination status, but it was not feasible to do this for the present study. However girls were offered the option of responding ‘don’t know’ if they were uncertain of their vaccination status. Although HPV vaccine coverage has increased since the programme began, uptake in London remains lower than the national average [3], which limits the generalizability of the findings of this study. The vaccine schedule and vaccine offered have changed since the study was conducted, however the findings of the present study are likely to remain relevant as girls are still required to receive a series of immunisations. Unfortunately, for the 16 % of girls who did not provide a reason for their vaccination status, we are unable to offer any further explanation. These data represent the reasons girls gave for remaining un-/under vaccinated, and while it is likely that there is concordance between parents’ and daughters’ reasoning, it is also possible that their parents might give may give different explanations. In the UK, parental consent for HPV vaccination is desirable, yet the vaccination can still be offered without it. At 12 years old girls are able to make their own decision if they are deemed competent to do so, and their understanding about HPV vaccination may also have implications for future cancer prevention behaviours. An unvaccinated girl may have to take more responsibility to have the vaccination in the future and to ensure she attends cervical screening. Girls’ attitudes may reflect their own opinions, or those of their parents.

Despite being part of a large study of almost 2,000 girls with a good overall response rate, the range of reasons given and our decision to present the findings by vaccine status means the numbers are too small to explore ethnic differences statistically. However we felt that it was meaningful to offer a descriptive analysis of the reasons given by different ethnic groups. We hope that these findings will give an indication of the themes that should be addressed in information about HPV vaccination and the reasons that may need to be considered when looking directly at un-/under vaccinated girls either across the population or if focussing on ethnic minority groups. Future research with larger samples will allow us to explore ethnic, as well as socioeconomic and geographical differences.

## Conclusions

Among girls from diverse ethnic backgrounds, practical problems are likely to inhibit girls from completing the HPV vaccination course, whereas safety concerns, and perceiving that the HPV vaccination is not necessary can explain why girls remain unvaccinated. The findings of this study may be used to tailor interventions to increase informed participation among girls who are currently under vaccinated or unvaccinated.

## Abbreviations

HPV: human papillomavirus
GP: general practitioner/family physician
PCT: primary care trust
FAS: family affluence scale
GCSE: general certificate of secondary education
FSME: Free School Meal Eligibility
